# Supplementary material for: Invasive Buttonweed Cotula coronopifolia (Asteraceae) Is Halotolerant and Has High Potential for Dispersal by Endozoochory
Source: Plants (Basel). 2024 Aug 10;13(16):2219. doi: 10.3390/plants13162219 (PMC11359061; doi:10.3390/plants13162219)
Supplement: Supplementary file 1 [file plants-13-02219-s001.zip › Suplementary_materials_v2.pdf]

SUPLEMENTARY MATERIAL

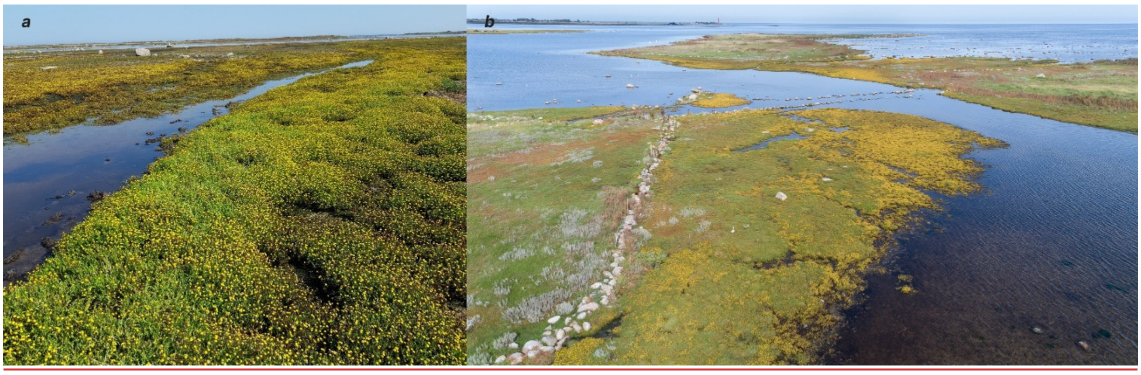

Figure S1. *Cotula coronopifolia* (yellow flowers) growing in Nedra Sandby on the east coast of Öland, Sweden where it has displaced native vegetation. This area is grazed by cattle and used by migratory waterbirds a) Photo taken in 2019 (by LT) and b) Drone photo taken in 2017 (photo credit: Thomas Gunnarsson).

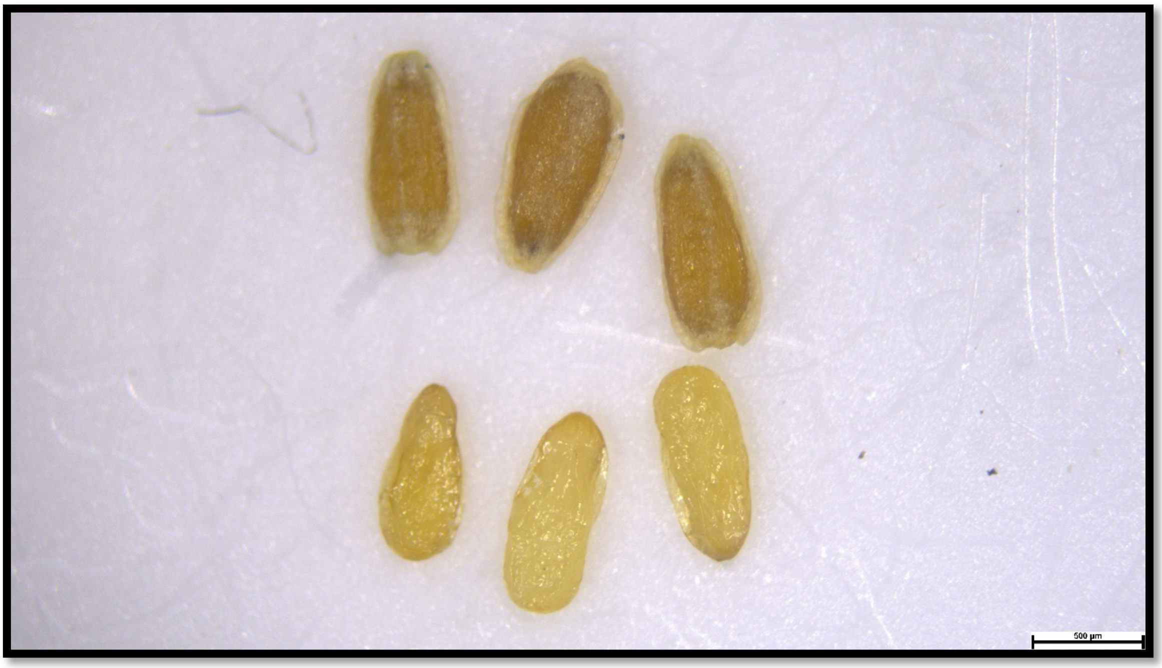

Figure S2. *Cotula coronopifolia* seeds before (top three seeds) and after (bottom three seeds) simulated gut passage by scarification and acid treatment. Photo credit: Iciar Jiménez Martín.

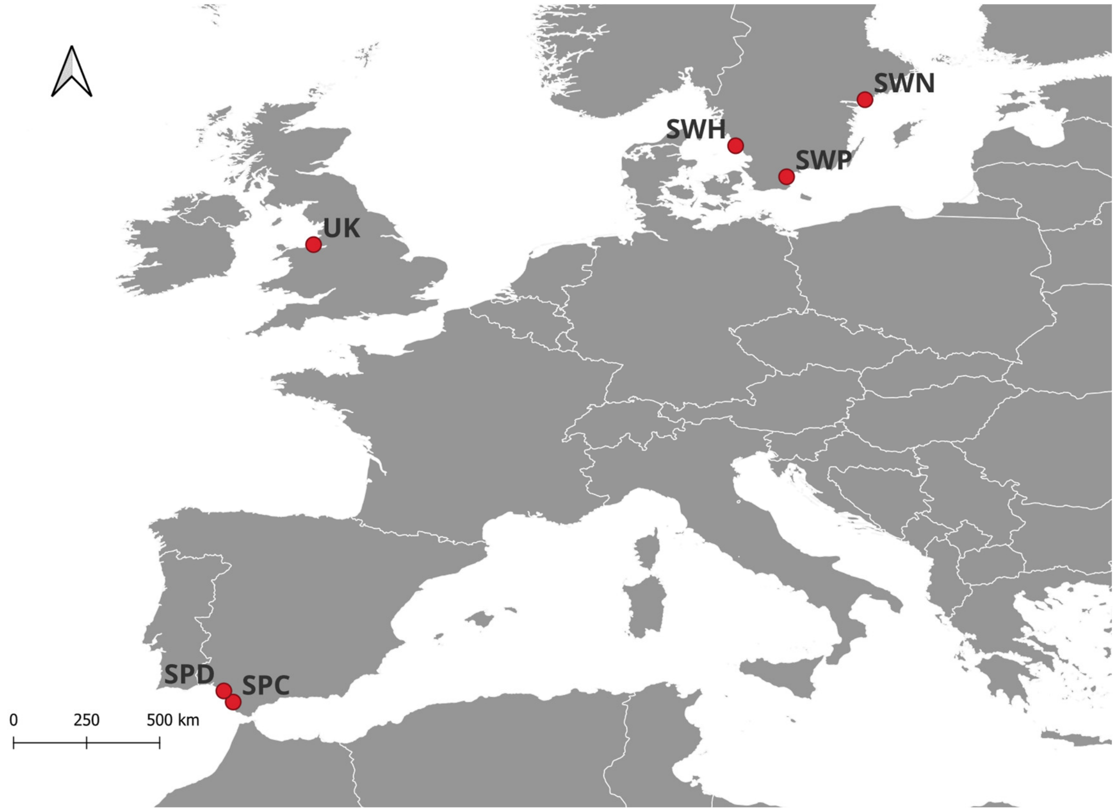

Figure S3. *Cotula coronopifolia* sampling sites. Red dots represent the GPS coordinates for each locality sampled for this experiment. Salinas de Cetina, Cádiz (SPC) and Laguna Dulce, Doñana (SPD) in the south of Spain; Halland (SWH), Nyköping (SWN) and Pulken (SWP) in Sweden; and Hoylake, Wirral (UK) in the United Kingdom.
